# Pantoprazole Decreases Cell Viability and Function of Human Osteoclasts *In Vitro*

**DOI:** 10.1155/2015/413097

**Published:** 2015-03-22

**Authors:** Markus Prause, Claudine Seeliger, Marina Unger, Elizabeth Rosado Balmayor, Martijn van Griensven, Alexander Tobias Haug

**Affiliations:** Experimental Trauma Surgery, Department of Trauma Surgery, Klinikum Rechts der Isar, Technical University Munich, Ismaninger Straße 22, 81675 Munich, Germany

## Abstract

Proton pump inhibitors (PPIs) are commonly prescribed drugs that decrease stomach acidity and are thus often used to treat gastroesophageal reflux disease and as a preventative agent for the adverse effects of nonsteroidal anti-inflammatory drugs on the stomach mucosa. In recently published literature, an association between proton pump inhibitor administration and increased fracture risk has been stated. In order to reveal the underlying pathomechanisms of these observations, the effects of pantoprazole, a representative of the proton pump inhibitors, on human osteoclasts *in vitro* were evaluated in this study. Osteoclasts were stimulated with increasing concentrations of pantoprazole ranging from 0 *μ*g/mL to 10 *μ*g/mL over a period of seven days. Cell viability and tartrate-resistant acid phosphatase (TRAP) activity assays were performed after 1 day, 3 days, and 7 days, respectively. Here, stimulated osteoclasts presented a significantly lower viability and TRAP activity than the negative controls. Osteoclast-specific gene expression was evaluated after seven days and revealed no significant differences between all samples. Overall, the bone degrading and resorptive function of osteoclasts is inhibited by the administration of proton pump inhibitors. While PPI-related fractures through “basic multicellular unit” dysfunction are unlikely, the underlying pathomechanism remains unknown.

## 1. Introduction

Pantoprazole is one of the most frequently used proton pump inhibitors (PPIs) in the clinical setting. By irreversibly inhibiting H+/K+ ATPase, or proton pump, in gastric cells, it strongly reduces the proton influx into the gastric lumen and therefore the effective stomach acidity. It is therefore used in the treatment of gastroesophageal reflux disease, in eradication treatment of* Helicobacter pylori* infection, and in combination with nonsteroidal anti-inflammatory drugs (NSAIDs) for prophylaxis of stress ulcers [1–3]. Although the administration of PPIs in a wide range of medical disciplines is necessary, they are often wrongly administered chronically and without a clear indication [4].

The administration of various PPIs has been shown to be significantly associated with an increased fracture risk, while a direct causal link between the two is not yet proven [5–7]. While short-term administration of PPIs already poses a moderately increased risk, long-term administration over a year or more seems to further increase the fracture risk [8, 9].

Insogna hypothesized a cell-independent mechanism why bone density should decrease under PPI therapy. According to his study, altered gastric acidity may lead to reduced calcium absorption, which would ultimately increase the rate of bone loss [10]. Similarly, Vestergaard et al. hypothesized a secondary hyperparathyroidism and subsequent osteoporosis as a possible cause for the increased fracture risk [11].

Our study group has previously investigated the influence of pantoprazole on osteoblasts* in vitro*. Osteoblasts represent a subpopulation of bone cells that synthesize the bone matrix. Pantoprazole seems to have beneficial effects on the viability of osteoblasts and their ability to synthesize new bone matrix [12]. The functional counterpart to osteoblasts within the basic multicellular units is osteoclasts, which actively degrade and resorb bone matrix. Together, these cellular units form a functional entity that secures dynamic bone homeostasis in order to meet the requirements of constant load transmission. It has been described that, additionally to their inhibitory effects on gastric proton pumps, PPIs may inhibit a proton pump in osteoclasts also known as the vacuolar ATPase [13]. It is mainly responsible for creating an acidic environment between the ruffled border of osteoclasts and bone tissue. In this acidic environment at the bone-apposed plasma membrane of the osteoclast, lytic enzymes are activated and bone is resorbed within the process of remodeling [14]. V-ATPases are related yet not identical to the proton pump that is located in parietal cells. Consequently, effects of PPIs on V-ATPases are less apparent than on gastric proton pumps [15].

Since no pathomechanism has yet been found that adequately explains or indeed proves a causal correlation between the use of PPIs and an increased fracture risk, the aim of this study was to investigate the effect of an important representative of the proton pump inhibitors—pantoprazole—on a cellular level.

## 2. Materials and Methods

The plastic labware used in our experiments was acquired from Sarstedt (Nümbrecht, Germany). The culture plates were acquired from GE Healthcare (Little Chalfont, UK). The chemicals were acquired from Sigma-Aldrich (St. Louis, MO, USA). Dentine chips were kindly provided by Associate Professor Susanne Mayer, MD, Laboratory for Biomechanics and Experimental Orthopaedics, Department of Orthopaedics, Ludwig-Maximilians-University, Munich, Germany.

### 2.1. Isolation of Peripheral Blood Mononuclear Cells (PBMCs)

Buffy coats (i.e., portions of donated blood that contain a concentrated amount of leucocytes) of male and female donors (*N* = 4) ranging from 50 to 67 years in age were obtained from Bayerisches Rotes Kreuz Blutspendedienst (Bavarian Red Cross Blood Donation Centre, Ulm, Germany). We selected donors of higher age since the prescription and therefore potential risks of PPIs are more imminent in older than in younger patients. After resuspension with 250 mL Dulbecco's phosphate buffered saline (DPBS) per buffy coat, 30 mL of suspension was layered on top of 20 mL lymphocyte separation medium (Biowest, Nuaillé, France) with a density of 1077 kg/m^3^. After 20 minutes of centrifugation at 1000 g without brakes, the PBMCs were isolated from the formed interphase. Afterwards, the cells were resuspended in DPBS and centrifuged again at 650 g for 10 minutes. This last step was repeated once more for purification. Finally, the PBMCs were resuspended in cultivation medium which consisted of 500 mL alpha modification minimum essential medium (PAA Laboratories GmbH, Cölbe, Germany), 10% fetal calf serum (FCS), 100 U/mL penicillin, and 10 *μ*g/mL streptomycin. The cells were then plated at a concentration of 3 million cells per well on 48-well plates for cell experiments and 6 million cells per well on 24-well plates for gene expression analysis.

### 2.2. Osteoclast Differentiation

24 hours after isolation, the isolation medium was exchanged for cultivation medium that contained 25 ng/mL macrophage colony-stimulating factor (M-CSF). All following media exchanges were performed on Mondays, Wednesdays, and Fridays. After a week of M-CSF stimulation, cultivation medium containing 12.5 ng/mL M-CSF and 10 ng/mL receptor activator of nuclear factor *κ*B (RANKL) was administered. For the following two weeks, the monocyte cultures were stimulated with medium containing 20 ng/mL RANKL.

### 2.3. Experimental Setup

After 24 days of differentiation, the osteoclasts were stimulated every other day with freshly prepared pantoprazole (PP) at the following concentrations: 0 *μ*g/mL (negative control), 0.1 *μ*g/mL, 1 *μ*g/mL, 3 *μ*g/mL, and 10 *μ*g/mL. These include the concentrations that correspond to the* in vivo* serum concentrations (1 *μ*g/mL and 3 *μ*g/mL) of the two most common prescription doses of pantoprazole (20 mg and 40 mg). This was done to reveal effects that are most relevant to clinical application. The effects of pantoprazole on osteoclast function, metabolism, and gene expression were analysed on days 1, 3, and 7 after the initial stimulation.

### 2.4. Osteoclast Phenotype: von Kossa Staining

In order to demonstrate and prove the osteoclastic phenotype and the ability to absorb mineralised matrix, PBMCs were plated on a culture plate coated with a thin layer of calcium phosphate (Corning, Tewksbury, MA, USA). After differentiation and stimulation, the medium was removed and the osteoclasts were incubated with NaClO for 5 minutes at room temperature. Afterwards, the wells were washed twice with dH_2_O and subsequently incubated with a 3% solution of silver nitrate in H_2_O for 30 minutes at room temperature. The silver nitrate solution was removed and the wells were washed repeatedly in dH_2_O over 10 minutes. Then, a 1% solution of pyrogallol in H_2_O was added and incubated for 3 minutes. After two additional washing steps with dH_2_O, the samples were fixated with a 5% solution of sodium thiosulfate in H_2_O for 5 minutes. After multiple washing steps with dH_2_O, pictures were taken using the BZ9000 fluorescence microscope (Keyence, Osaka, OSK, Japan). Through the von Kossa staining, the remaining calcium phosphate appears as a dark grey and the resorption pits appear as a light grey to white shade.

### 2.5. Osteoclast Phenotype: Toluidine Blue Staining

Toluidine blue staining was performed on osteoclasts that were cultivated on dentine chips in suspension culture plates to show the formation of resorption lacunae. The cells were lysed with sodium hypochlorite solution and wiped off the dentine chips with a paper towel. After short immersion in 1% toluidine blue solution, pictures were taken with the BZ9000 fluorescence microscope (Keyence, Osaka, OSK, Japan).

### 2.6. Osteoclast Phenotype: TRAP Staining

To visualise the presence and resorptive activity of osteoclasts and thus prove the successful differentiation, TRAP staining was performed on control samples after 1 day, 3 days, and 7 days. The cells were fixated with a solution of 10% DPBS, 4% formaldehyde, and 0.2% Triton X-100 in dH_2_O for 5 minutes. The staining buffer consisted of 40 mM sodium acetate and 10 mM disodium tartrate dihydrate in ddH_2_O (pH = 5). 0.01% naphthol AS-MX phosphate, 0.06% Fast Red Violet LB salt, and 1% N-N dimethylformamide were added to get the final staining solution. After the fixation, the cells were air-dried at room temperature. Finally, the staining solution was added and incubated for 20 minutes at 37°C. After a washing step with DPBS, pictures were taken using the BZ-9000 fluorescence microscope (Keyence, Osaka, OSK, Japan).

### 2.7. TRAP Activity

In order to determine the level of osteoclast resorption activity, the TRAP activity of the osteoclasts was assessed. The assay buffer consisted of 100 mM sodium acetate and 50 mM disodium tartrate dihydrate in ddH_2_O (pH 5.5). The substrate buffer solution consisted of 5 mM 4-nitrophenyl phosphate disodium salt hexahydrate in assay buffer. For TRAP activity analysis, 50 *μ*L of cell supernatant of each sample was transferred to a 96-well plate. The aliquots were incubated at 37°C with 150 *μ*g/mL assay buffer per well for 1 hour. A standard curve was prepared by stepwise dilution of 1 mM 4-nitrophenol in a 1 : 4 solution of osteoclast culture medium and assay buffer. After the total incubation time, the reaction was stopped using 50 *μ*L of 3 M NaOH solution. Finally, the absorbance was detected at *λ* = 405 nm. TRAP activity was calculated according to the standard curve.

### 2.8. 3-(4,5-Dimethylthiazol-2-yl)-2,5-diphenyltetrazolium Bromide (MTT) Assay

In order to quantify cell viability, the MTT assay was performed and set in relation to the negative control. The samples were incubated with 1.2 mM thiazolyl blue solution for 120 minutes at 37°C and 5% CO_2_. Afterwards, the supernatant was removed and the remaining formazan crystals were resolved in a dimethyl sulfoxide solution containing 10% sodium dodecyl sulfate and 0.6% acetic acid. The formazan content was determined through photometrical measurements at *λ* = 570 nm and *λ* = 690 nm.

### 2.9. Gene Expression

In order to evaluate gene expression activity, semiquantitative analyses of reverse transcription polymerase chain reaction (RT-PCR) products were performed. The total RNA was isolated from the cells using TriFast (Sigma-Aldrich, St. Louis, MO, United States), phenol chloroform extraction, and subsequent ethanol precipitation. The RNA was transcribed into complementary DNA (cDNA) using the First-Strand cDNA Synthesis Kit (Fermentas, St. Leon-Rot, Germany). RT-PCRs were performed after a standard protocol. The respective primers of the investigated genes are listed in Table 1. Ethidium bromide was used for visualisation of the PCR products that were separated in 1.8% w/v agarose gels. The densitometric analysis of the generated signals was performed with the ImageJ software (NIH, Bethesda, MD, USA). The housekeeping gene *β*-actin (*ACTB*) was used for normalisation.

## 3. Statistics

The experiments were performed independently on four individual specimens (*N* = 4). The measurements were performed as duplicates or more (*n* ≥ 2). The gathered results are depicted as mean ± SEM. Kruskal-Wallis test and Dunn's multiple comparisons test (GraphPad Prism Software, El Camino Real, USA) were used for data comparison. The minimal value of significance was *P* < 0.05.

## 4. Results

### 4.1. von Kossa Staining/Toluidine Blue Staining/TRAP Staining

**Figure 1:**
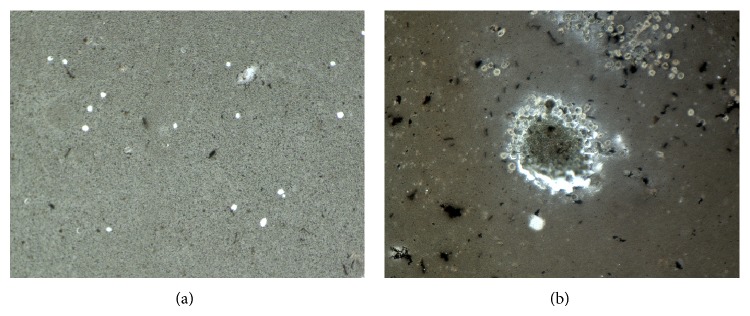
Depicted are two exemplary fields of view of the von Kossa-stained calcium phosphate plate. The pictures were taken of samples that were not stimulated with pantoprazole, 7 days after differentiation. Multiple resorption zones (white spots) where osteoclasts were located prior to lysis are visible at 20x magnification (a). A large cluster of osteoclasts is visible in the middle of the picture with several smaller clusters in the periphery of the picture at 10x magnification (b).

**Figure 2:**
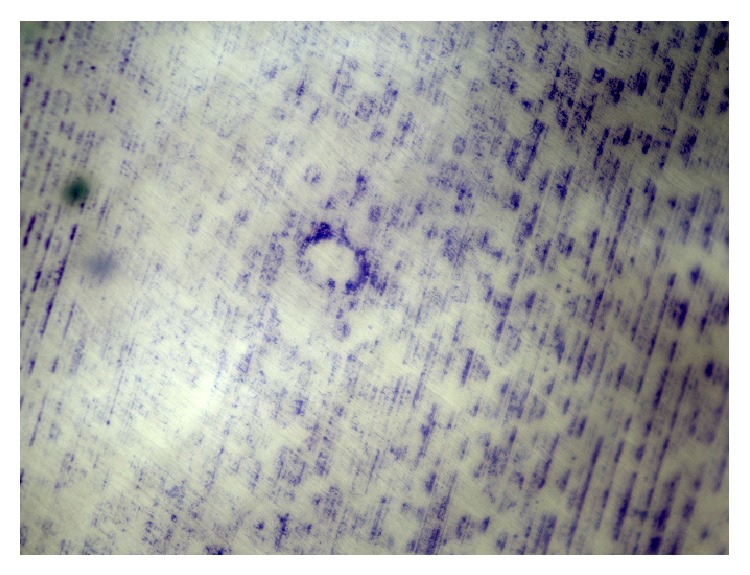
A resorption lacuna is made visible on the dentine chip through toluidine blue staining. The picture was taken under light microscopy at 10x magnification, 7 days after differentiation. The sample in this picture consisted of osteoclasts that were not stimulated with pantoprazole.

**Figure 3:**
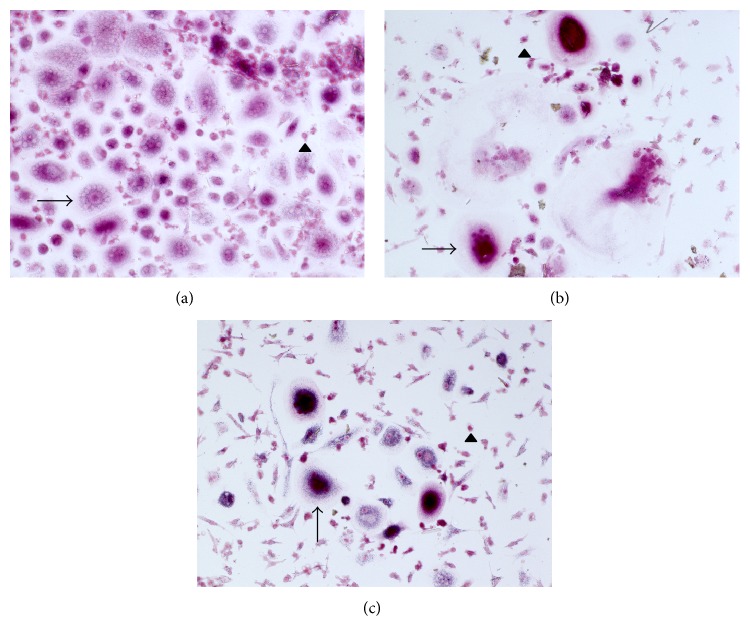
Depicted are three representative fields of view of TRAP-stained samples, 1 day (a), 3 days (b), and 7 (c) days after differentiation and without pantoprazole stimulation. Visible are multinucleated osteoclasts (arrows) as well as mononuclear cells that have not yet merged (arrowheads).

The stainings were performed on negative control samples of each donor 7 days after completed differentiation. The von Kossa-stained samples show that the differentiated cells actively resorbed calcium phosphate (Figures 1(a) and 1(b)) while the exemplary toluidine blue-stained dentine chip shows the formation of Howship's lacuna (Figure 2). Furthermore, the strong accumulation of TRAP stain in the depicted cells suggests successful differentiation of the PBMCs into active osteoclasts after days 1, 3, and 7 after differentiation (Figures 3(a), 3(b), and 3(c)).

### 4.2. TRAP Activity

**Figure 4:**
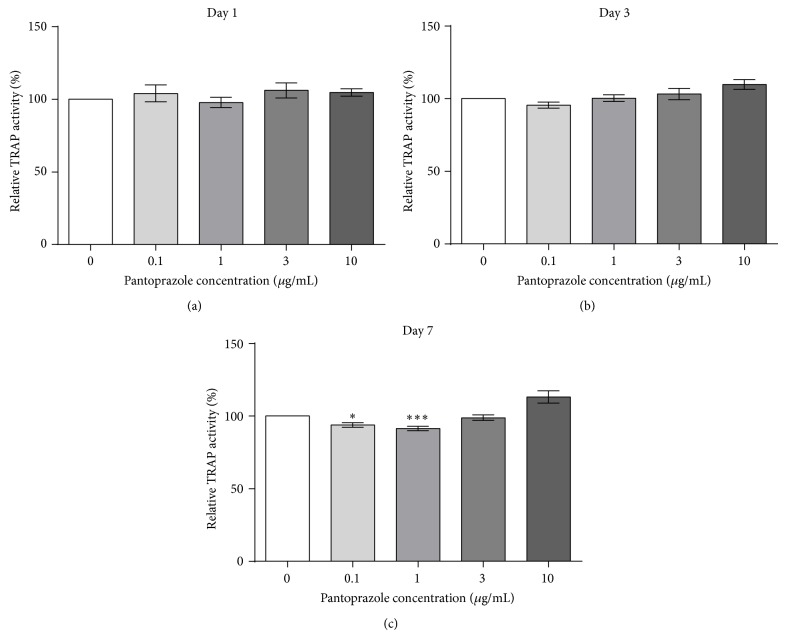
The relative TRAP activity of osteoclast samples (*N* = 4, *n* = 6), that is, the activity of samples stimulated with pantoprazole normalized to untreated samples, was assessed on days 1, 3, and 7 of stimulation. The results were accordingly expressed as a percentage of the negative control. Shown are column bar graphs of means ± SEM. ^*^ *P* < 0.05 and ^***^ *P* < 0.001 versus the untreated controls.

TRAP activity assessed on days 1 and 3 after initial differentiation showed no significant differences between the control and samples stimulated with PP (Figures 4(a) and 4(b)). On day 7, however, the TRAP activity of the 0.1 *μ*g/mL and 1 *μ*g/mL PP groups showed a decrease of 6.12% (*P* < 0.05) and 8.56% (*P* < 0.01), respectively (Figure 4(c)).

### 4.3. MTT Assay

**Figure 5:**
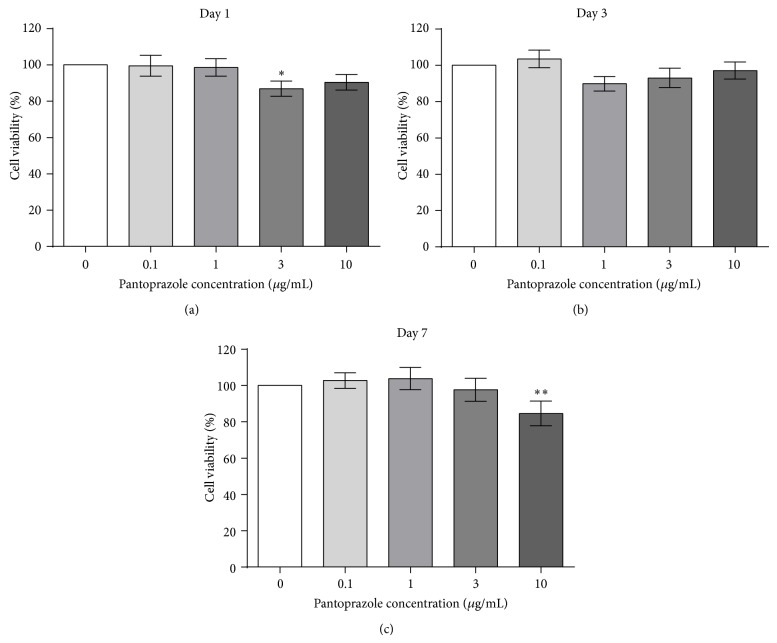
The MTT cell viability assays performed on days 1, 3, and 7 of stimulation with pantoprazole (*N* = 4, *n* = 6). The viability in relation to the respective negative control was assessed. The results were expressed as percentages of the negative control values. Shown are column bar graphs of means ± SEM. ^*^ *P* < 0.05 and ^**^ *P* < 0.01 versus the untreated controls.

The performed MTT assay revealed a decreased viability in osteoclast cultures stimulated with PP. On day 1, the viability of the samples exposed to 3 *μ*g/mL PP was decreased by 13.04% (*P* < 0.05). The viability of the other sample groups decreased as well, yet without statistical significance (Figure 5(a)). On day 3, stimulation with 1 *μ*g/mL PP or more led to a decreased viability and was also statistically insignificant (Figure 5(b)). On day 7, the viability of samples that were stimulated with 10 *μ*g/mL PP was decreased by 15.33% (*P* < 0.01) (Figure 5(c)).

### 4.4. Gene Expression

**Figure 6:**
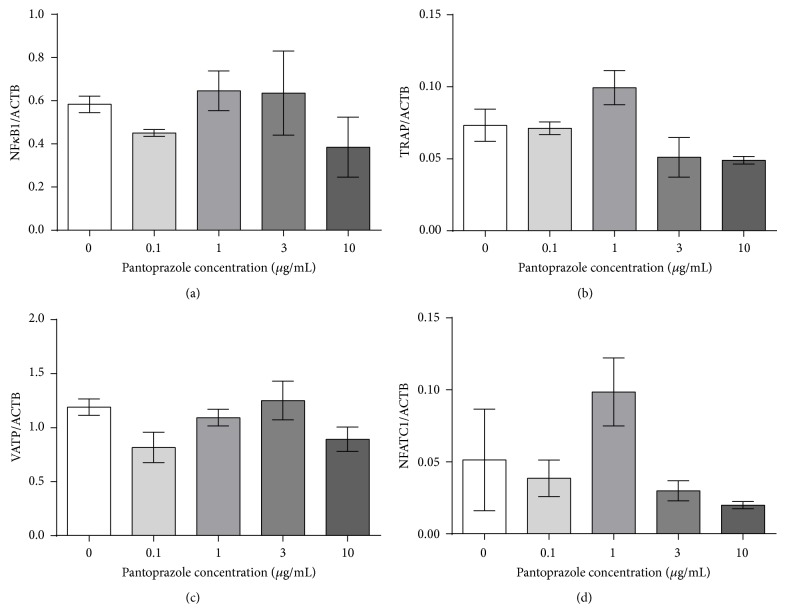
Gene expression analysis of* NFκB* (a),* TRAP* (b), VATP (c), and* NFATC1* (d) was performed after 7 days of stimulation with pantoprazole (*N* = 4, *n* = 2). The results were normalized to the housekeeping gene *β*-actin (ACTB). Shown are column bar graphs of means ± SEM.

The gene expression analysis of nuclear factor kappa-light-chain-enhancer of activated B cells (*NFκB*), tartrate-resistant acid phosphatase (*TRAP*), a subunit of the V-ATPase (*VATP*), and nuclear factor of activated T-cells, cytoplasmic 1 (*NFATc1*), revealed no significant differences after 7 days of stimulation (Figures 6(a), 6(b), 6(c), and 6(d)).

## 5. Discussion

The question whether PPIs have detrimental effects on bone metabolism and may in fact cause an increased fracture risk, as well as being a risk factor for other pathologies, such as community-acquired pneumonia, is of great interest in the clinical setting [5, 6, 8, 16, 17]. The fact that most standard pain management therapies include the concomitant administration of PPIs, in many cases over long periods of time, would make negative effects particularly harmful. In this study, we explored the effects of pantoprazole, one commonly prescribed PPI, on a cellular level.

After successful initial differentiation of the isolated PBMCs into osteoclasts, our stimulation experiments were carried out over a period of 7 days. The performed cell viability assays, as well as the TRAP activity assays, suggest that human osteoclasts are significantly inhibited in their overall ability to degrade and absorb bone matrix. This is in accordance with the findings of Sheraly et al., who showed in their* in vivo* experiments that pantoprazole (0.5 mg/mL) and omeprazole (40 mg/mL) decrease the resorptive activity of osteoclasts when instilled into calcium phosphate cement [18]. Additionally, Hyun et al., who investigated the effects of omeprazole on murine osteoclast-like RAW 264.7 cells, revealed that osteoclast function is overall inhibited, while osteoblast function is enhanced, leading to an osteopetroricket-like bone matrix [19]. In synopsis of our studies on human osteoblasts and osteoclasts, as well as various studies that confirm the inhibitory effects of PPI on osteoclast function, the development of osteoporosis through effects on a cellular level as a causal link between PPI treatment and an increased fracture risk seems unlikely [6, 12, 20]. Accordingly, Targownik et al., who used a multivariate linear regression model on a set of data from the Canadian Multicentre Osteoporosis Study to determine whether there is a relevant decrease in bone mineral density (BMD) after administration of PPIs, revealed that there was no association between PPI usage and accelerated BMD loss over a ten-year period, although PPI users had a lower average baseline BMD [21].

However, not all studies agree on the effects of PPIs on bone homeostasis. In an early study by Mizunashi et al., for example, the administration of omeprazole led to an increase in serum levels of parathyroid hormone and subsequently TRAP, alkaline phosphatase, and bone glia protein (osteocalcin) while decreasing urinary excretion of calcium. This was thought to suggest overall increased bone turnover [22]. Costa-Rodrigues et al. on the other hand proposed in their study that there is the possibility of a dose-dependent decrease in bone turnover with the administration of PPIs [23]. Finally, several meta-analyses that were performed on recent observational studies hypothesized that the heterogeneity of results concerning the increased fracture risk could be due to hitherto unknown confounders and called for further investigation [6, 7].

## 6. Conclusions

PPIs are commonly used in combination with NSAID in pain management regiments for patients with bone-related pathologies. An increased fracture risk implying adverse effects on bone homeostasis would especially limit the safe application of this group of drugs as first line therapy in this context. In synopsis of this and several other* in vitro* studies, we think that the development of osteoporosis through direct cellular impairment is unlikely. Under PPI stimulation, the ability of osteoclasts to resorb mineralized matrix seems to be inhibited, while the ability of osteoblasts to synthesize new matrix is increased.

Reduced bone turnover as a result of the decreased resorption rate could potentially contribute to an increased fracture risk, as the bone matrix is not optimally adapted to its constantly changing load. Additionally, cell-independent causes of osteoporosis, for example, decreased calcium absorption or increased parathyroid hormone excretion, remain possible explanations for the association between PPI use and fracture risk. Finally, it must also be considered that the increased fracture risk found in observational studies is not causally related to the administration of PPIs but rather caused by an unknown confounder and therefore factually nonexistent. To prove a causal link between the use of PPIs and an increased fracture risk, further investigations into potential confounding factors as well as* in vivo* studies to further illustrate effects of the drugs on the cells in their natural environment are necessary.

## Figures and Tables

**Table 1:** Primers used for RT-PCR analysis.

Gene	Forward primer (5′-3′)	Reverse primer (5′-3′)
*ACTB *	ACAGAGCCTCGCCTTTGCCGAT	GCGAAGCCGGCCTTGCACAT
*NFκB*	GCGCCGCTTAGGAGGGAG	GAAGGTATGGGCCATCTGCTGTT
*TRAP *	CCCTCGGAGAAACTGCATCAT	CATGTCCATCCAGGGGGAGA
*VATP *	GGGCTGCTCATGTTCCTCTT	CACGTGGTTGAAGACTCCGA
*NFATC1 *	GCTTTAAAAAGGCAGGAGGCA	GAGGAAAGTCATCGAGGGGC
